# Adverse Perinatal Outcomes in COVID-19 Infected Pregnant Women: A Systematic Review and Meta-Analysis

**DOI:** 10.3390/healthcare10020203

**Published:** 2022-01-20

**Authors:** Malshani L. Pathirathna, Buddhini P. P. Samarasekara, Thakshila S. Dasanayake, Padmapriya Saravanakumar, Ishanka Weerasekara

**Affiliations:** 1 Department of Nursing, Faculty of Allied Health Sciences, University of Peradeniya, Kandy 20400, Sri Lanka; sdbppsamarasekara@gmail.com (B.P.P.S.); thakshila760@gmail.com (T.S.D.); 2 School of Clinical Sciences, Auckland University of Technology, Auckland 1142, New Zealand; Priya.Saravanakumar@aut.ac.nz; 3 Department of Physiotherapy, Faculty of Allied Health Sciences, University of Peradeniya, Kandy 20400, Sri Lanka; rmimw@pdn.ac.lk; 4 College of Health Medicine and Wellbeing, The University of Newcastle, Newcastle 2308, Australia

**Keywords:** COVID-19, perinatal outcomes, systematic review

## Abstract

The impact of COVID-19 virus infection during pregnancy is still unclear. This systematic review and meta-analysis aimed to quantitatively pool the evidence on impact of COVID-19 infection on perinatal outcomes. Databases of Medline, Embase, and Cochrane library were searched using the keywords related to COVID-19 and perinatal outcomes from December 2019 to 30 June 2021. Observational studies comparing the perinatal outcomes of COVID-19 infection in pregnancy with a non-infected comparator were included. The screening process and quality assessment of the included studies were performed independently by two reviewers. Meta-analyses were used to pool the comparative dichotomous data on perinatal outcomes. The database search yielded 4049 results, 1254 of which were duplicates. We included a total of 21 observational studies that assessed the adverse perinatal outcomes with COVID-19 infection. The odds of maternal death (pooled OR: 7.05 [2.41−20.65]), preeclampsia (pooled OR: 1.39 [1.29−1.50]), cesarean delivery (pooled OR: 1.67 [1.29−2.15]), fetal distress (pooled OR: 1.66 [1.35−2.05]), preterm birth (pooled OR: 1.86 [1.34−2.58]), low birth weight (pooled OR: 1.69 [1.35−2.11]), stillbirth (pooled OR: 1.46 [1.16−1.85]), 5th minute Apgar score of less than 7 (pooled OR: 1.44 [1.11−1.86]) and admissions to neonatal intensive care unit (pooled OR: 2.12 [1.36−3.32]) were higher among COVID-19 infected pregnant women compared to non-infected pregnant women.

## 1. Introduction

Coronavirus disease 2019 (COVID-19), caused by the SARS-CoV-2 virus, continues to be an alarming global public health crisis [1] with a sharply escalating number of deaths that have largely surpassed previous fatalities caused by epidemics such as Middle Eastern Respiratory Syndrome (MERS) and Severe Acute Respiratory Syndrome (SARS) [2]. At the time of writing (3 December 2021), 263,563,622 confirmed cases of COVID-19, including 5,232,562 deaths, had been reported to the World Health Organization (WHO) [3]. This situation raises concerns in vulnerable populations such as pregnant mothers, fetuses and their neonates. Pregnant women are at higher risk of developing severe illness from respiratory infections, largely due to immunodeficiency associated with physiological adaptations during pregnancy [4]. Respiratory infections could escalate rapidly to respiratory failure, leading to potentially fatal consequences for both mother and fetus [5]. A recent multinational retrospective cohort study of 388 pregnant women reported that SARS-CoV-2 infected pregnant women risk fatal consequences from compromised respiratory functions and need intensive care [6]. Healthcare systems continue to become over-burdened, risking compromised access and quality of services. Maternal and child health services are no exceptions to these challenges. Furthermore, low awareness of prevention strategies [7], mixed information from the COVID-19 infodemic [8], scarcity of healthcare, and intensive care services [9] accentuate the negative effects on the populations. There has been a steep rise in publications, including literature reviews on COVID-19 in pregnancy globally. However, the quality of several studies has been varied, with some including case reports and case series [10,11] and several reviews becoming outdated with the emergence of new evidence. Scientifically proven up-to-date evidence of maternal, fetal, and neonatal risks associated with COVID-19 infection in pregnancy is an urgent need to guide clinical decision-making in maternal and child health care. Hence, we conducted this systematic review on adverse perinatal outcomes in COVID-19 infected mothers. Our primary aim was to evaluate the maternal, fetal and neonatal effects associated with COVID-19 in pregnancy. As secondary aims, we evaluated the incidence of COVID-19 among pregnant women and the comorbidity profiles of COVID-19 infected pregnant women. Knowledge of the effects of COVID-19 on pregnancy, childbirth, and postpartum is essential for maternal health care service providers to plan effective management strategies. The prevalence of COVID-19 related adverse perinatal outcomes and the comorbidity profiles of COVID-19 infected pregnant women are essential variables that would help inform care and preventative services.

## 2. Materials and Methods

We conducted this systematic review and meta-analysis based on the PROSPERO protocol registered on 18 May 2021 (CRD42021254974). This review included studies focused on perinatal outcomes of COVID-19 infection in pregnancy, mainly to evaluate the reported adverse perinatal outcomes in COVID 19 infected mothers and the prevalence of adverse perinatal (maternal, fetal, and newborn) outcomes in COVID-19 infected pregnant women. This review reports adverse maternal, fetal, and newborn outcomes of COVID-19 infected pregnant women, comorbidities in COVID-19 infected pregnant women, and the incidence of COVID-19 infection among pregnant women in line with the updated PRISMA 2020 guidelines for reporting systematic reviews [12].

### 2.1. Eligibility Criteria, Data Sources and Search Strategy

Observational studies (cohort and case–control) investigating the perinatal outcomes in COVID-19 infected pregnant women and published as peer-reviewed articles in English were eligible for inclusion. Case reports, case series, editorials, letters to the editor, perspectives, conference papers, narrative or systematic reviews, and studies without a non-infected pregnant group as the comparator were excluded. We searched Medline, EMBASE, and Cochrane Library databases to identify the published studies from December 2019 to 30 June 2021. The search strategy included a combination of keywords for COVID-19 and perinatal outcomes (Table S1).

### 2.2. Study Selection

All of the identified studies from the database search were exported to EndNote reference management software (version EndNote X9.3.3.). Then, Covidence systematic review software (Veritas Health Innovation, Melbourne, Australia) was used to manage the independent screening process at both the stages of title and abstract screening (M.L.P., B.P.P.S., T.S.D.) and full-text screening (M.L.P., B.P.P.S., T.S.D.). Reasons for full-text exclusion were documented at the full-text screening stage. Any disagreement was resolved by consensus or by consultation with a third reviewer at both stages.

### 2.3. Data Extraction

The data from the included studies were extracted to an Excel sheet by one author, and another author cross-checked the accuracy. The extracted data included the study characteristics (country, year of publication, study design and methodology, study period, population and setting, total number of participants, number of cases, number in control group and drop-outs), participants’ socio-demographic and baseline data, comorbidities, adverse perinatal outcomes (maternal death, termination of pregnancy, miscarriage or abortion, preeclampsia, pre-labor rupture of membrane (PROM), preterm pre-labor rupture of membrane (PPROM), intrauterine death, fetal distress, preterm birth, low birth weight, stillbirth, Apgar score, admissions to neonatal intensive care unit (NICU), neonatal deaths, cesarean section deliveries, and operative vaginal births, the incidence of COVID-19 among pregnant women, and the outcome of interest of each study.

### 2.4. Assessment of Risk of Bias

Quality assessments of the included studies were performed using the National Institute of Health’s (NIH) study quality assessment tool for observational, cohort, and cross-sectional studies and the NIH study quality assessment tool for case–control studies [13]. The quality of each study was independently assessed by two assessors (B.P.P.S. and T.S.D). Any disagreement was resolved through consensus between the two assessors.

### 2.5. Data Synthesis and Analysis

The characteristics of the included studies, characteristics of the COVID-19 infected pregnant women and the summary findings were tabulated. Further, the incidence of COVID-19 among pregnant women was graphically presented. Quantitative meta-analysis was carried out to pool the comparative dichotomous data of perinatal outcomes when more than one study presented the data for the relevant outcome. If individual studies reported no adverse outcome in the infected group or non-infected group, they were excluded from the meta-analysis of that particular perinatal outcome. Heterogeneity of studies was determined using the I^2^ statistic, where substantial heterogeneity was defined as I^2^ ≥ 30. Random effects estimates of the pooled odds of each perinatal outcome and comorbidity condition were generated using the Mantel–Haenszel method. The findings of each outcome comparison were summarized with odds ratio, 95% confidence interval, *p*-value, and the I^2^ statistic. Funnel plots were generated to visually evaluate the presence of publication bias.

## 3. Results

### 3.1. Study Selection

**Figure 1:**
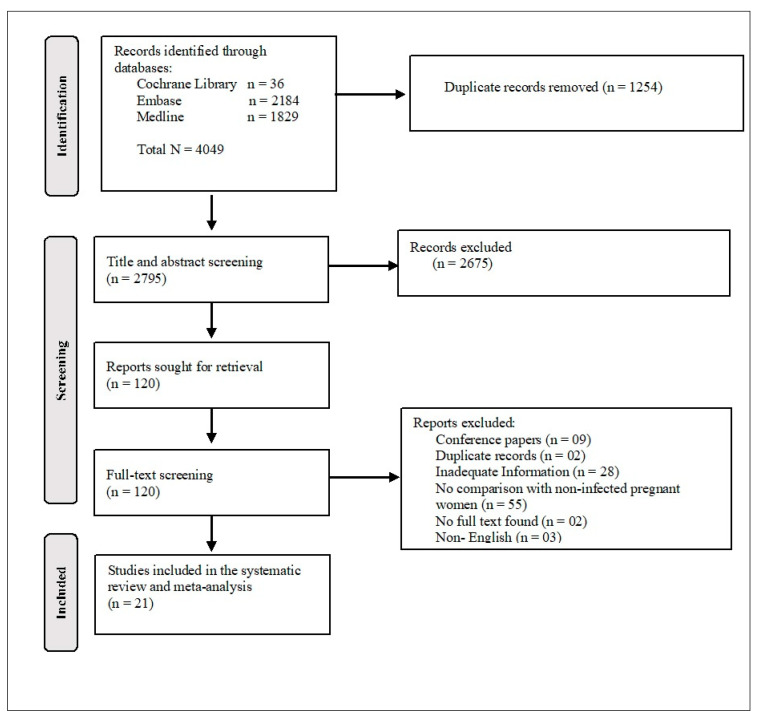
PRISMA flow chart of included studies.

Two thousand seven hundred ninety-five (2795) studies were identified through the search engines for the title and abstract reviews after removing 1254 duplicates. Out of the total screened abstracts, 120 were selected for full-text screening, of which 99 studies were excluded (mainly due to lack of a comparison group or the presentation of inadequate data), and 21 [14,15,16,17,18,19,20,21,22,23,24,25,26,27,28,29,30,31,32,33,34,35,36] studies were included in this systematic review and meta-analysis (Figure 1).

### 3.2. Study Characteristics

There were nine (42.9%) articles from single-center studies [14,15,16,17,20,21,22,25,27], eight (38.1%) from multicenter studies [18,19,23,24,26,29,31,32] and three (14.3%) from nationwide [28,30,34] studies. The remaining one (4.7%) was a multinational study [33]. Of the 20 studies included except the multinational study, eight (40%) were from the United States of America (USA) [15,16,20,23,24,25,29,31] three (15%) were from Spain [18,19,26], two (10%) each from Mexico [17,30] and India [21,27] and one (5%) each from Iran [14], United Kingdom (UK) [34], France [22], Sweden [28] and Canada [32]. Among the total included studies, 18 (85.7%) used cohort study design [14,15,18,19,20,21,22,24,25,26,27,28,29,30,31,32,33], while one cohort study used a historical comparison cohort [34]. Table 1 shows the characteristics of the included 21 studies.

### 3.3. Risk of Bias of Included Studies

**Figure 2:**
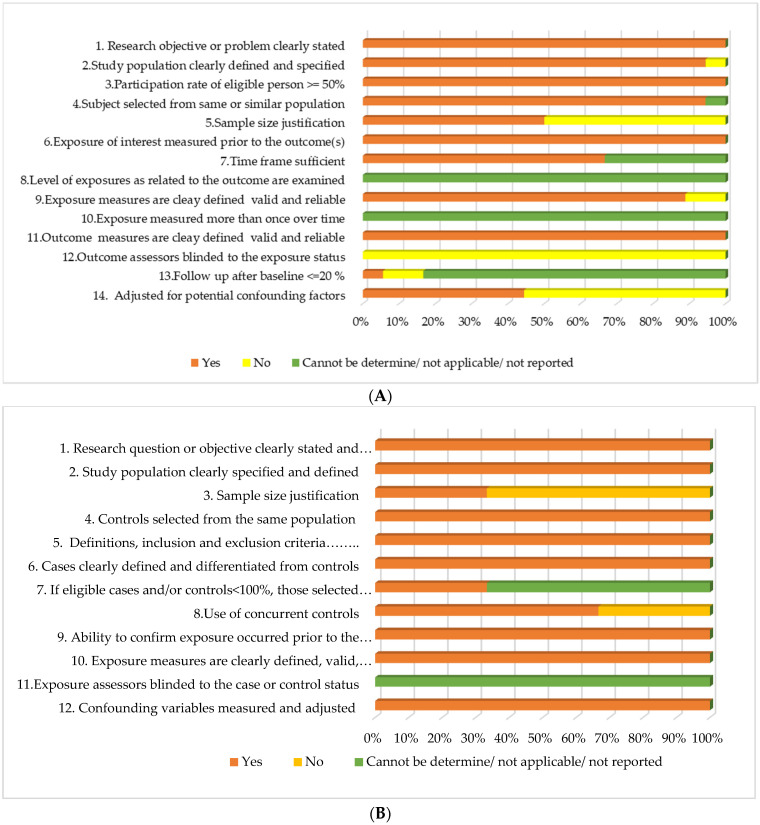
(**A**) Quality assessment of included cohort studies; (**B**) Quality assessment of included case–control studies.

Regarding the quality of the included cohort studies, 10 criteria out of 14 (71%) were satisfied by 40% of the included studies. Almost all of the studies had clearly stated research objectives, clearly defined study populations, clearly defined valid and reliable outcomes, and over ≥50% participation rate by eligible persons. In almost all the included studies, the quality assessment was unable to determine the level of exposures related to examined outcomes, exposure measures more than once over time, and follow-up after baseline. Blinding of the assessors to the exposure status was a serious concern for all the included studies. Only 40–55% of the included cohort studies were marked positively for the criteria of adjusting for potential confounding factors and having a justified sample size. With regards to the quality of included case–control studies, eight criteria out of 12 (75%) were satisfied by 60% of the included studies. All the studies satisfied the criteria related to clearly defined objective/s, clearly defined study population, selection of the control from the same population, consistent use of defined inclusion and exclusion criteria, clearly defined and differentiated case and control groups, ability to confirm the exposure occurred prior to the development of the condition, implementation of valid and reliable exposure measures, and measuring and adjusting for confounding variables. Blinding of the assessors to the exposure status was not determinable in all the studies. Less than 35% of the included case–control studies had a justified sample size (Figure 2A,B). Individual study assessments were attached as a supplementary file (Table S2).

### 3.4. Incidence of COVID-19 Infection in Pregnant Women

**Figure 3:**
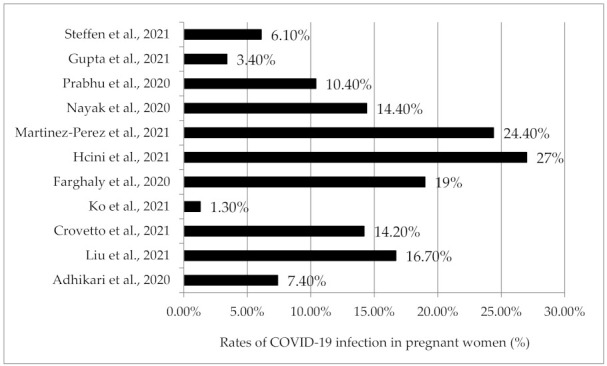
Incidence of COVID-19 among pregnant women.

Eleven studies reported the incidence of COVID-19 among pregnant women, with rates ranging from 1.3% to 27%. Only cohort studies were used to determine the incidence of COVID-19 infection in pregnant women. Even though there were 18 cohort studies, a few did not report the total number of admissions, making it difficult to quantify the incidence. Among the 11 that reported incidence, there were six studies from the USA [15,20,24,25,29,31], three single-center [15,20,25] and three multicenter studies [24,29,31]. The reported rates in the USA ranged from 1.3% to 19%. The highest rate (27%) of COVID-19 in pregnancy was reported from a single-center study conducted in France [22], while the second-highest rate was noted from a multicenter study conducted in Spain [26] (Figure 3).

### 3.5. Characteristics of COVID-19 Infected Pregnant Women

In the 21 included studies, a total of 14,131 COVID-19 infected pregnant women were studied compared to 585,376 COVID-19 non-infected pregnant women. The reported mean age of infected pregnant women ranged from 24.7 to 32.6 years, while some of the studies reported median (IQR) values ranging from 25 (21–31) to 33.3 (29–37) years (Table 2).

### 3.6. Summary Findings of Included Individual Studies

Out of 21 studies, six reported that COVID-19 infection during pregnancy was not associated with adverse perinatal outcome [15,18,25,30,31,32]. A study conducted in Spain concluded that even with no difference in the overall rate of adverse perinatal outcomes among COVID -19 infected women, symptomatic status was associated with a modest increase in preterm delivery and intrapartum fetal distress [18]. All of the other studies reported one or more significant adverse perinatal outcomes associated with COVID-19 in pregnancy. Table 3 shows the summary findings of individual studies included in this systematic review.

### 3.7. Adverse Perinatal Outcomes of COVID-19 Infection in Pregnancy

#### 3.7.1. Adverse Maternal Outcomes

**Figure 4:**
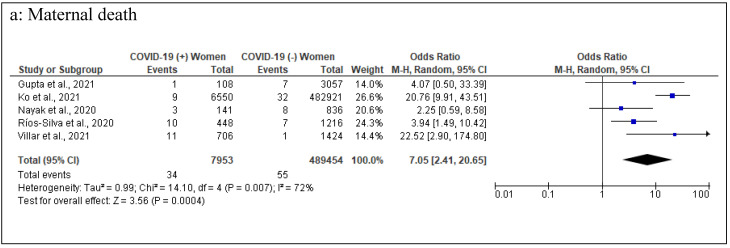

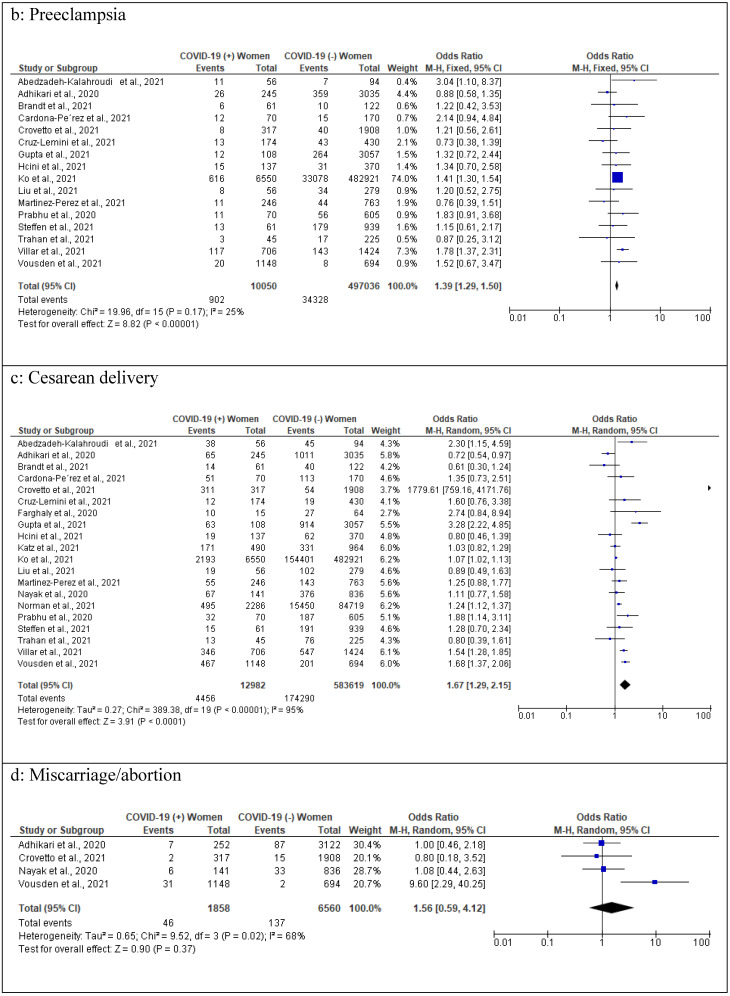

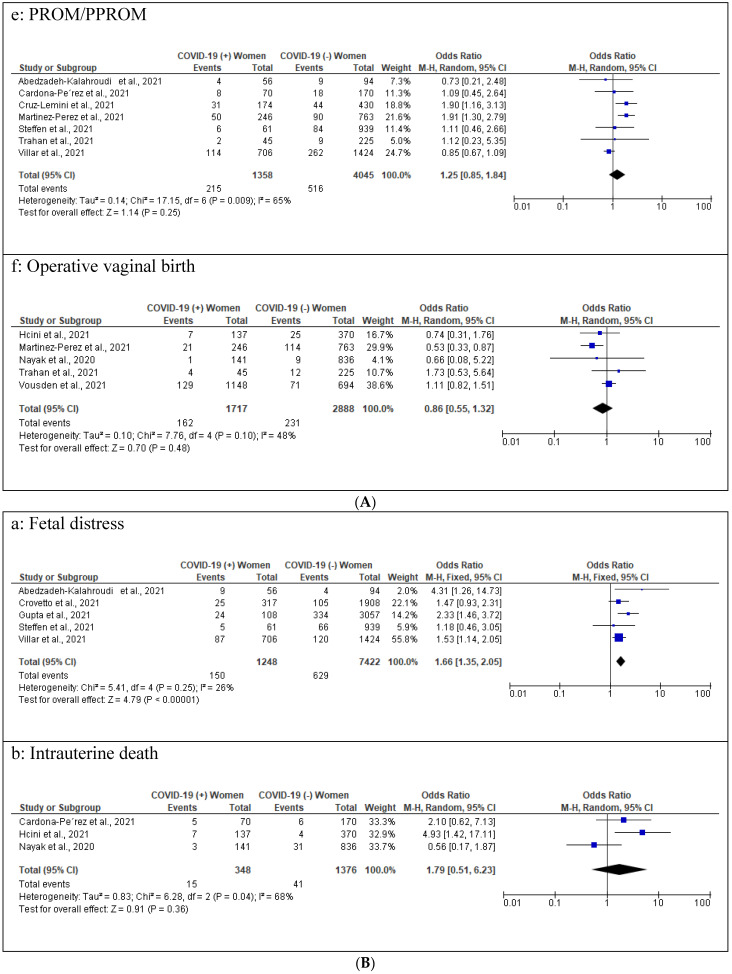

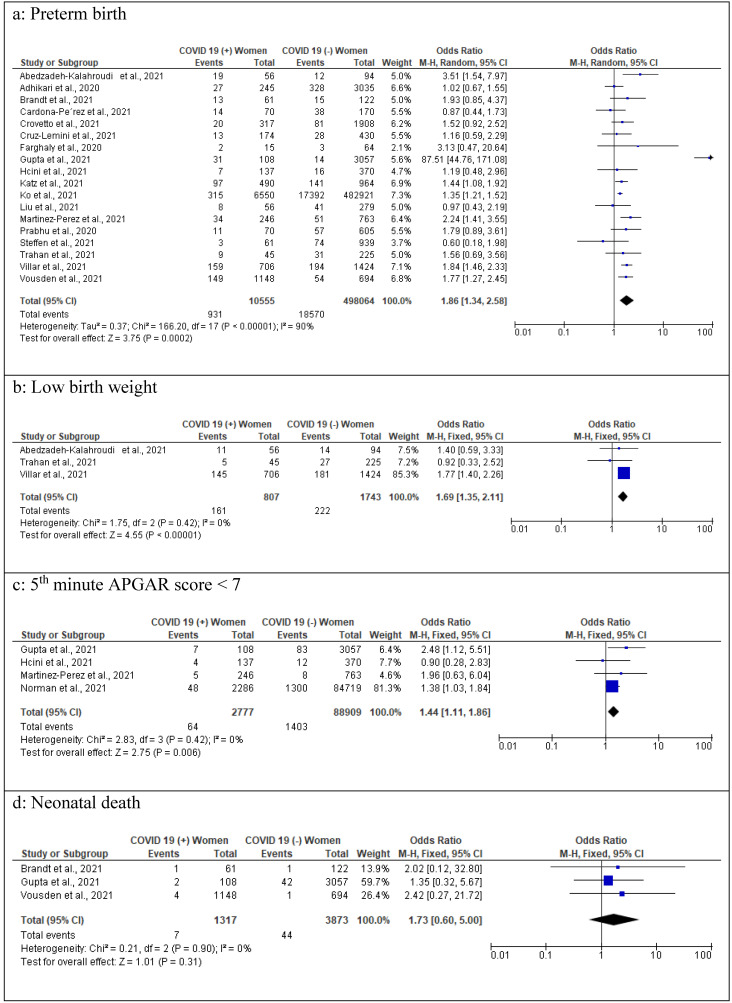

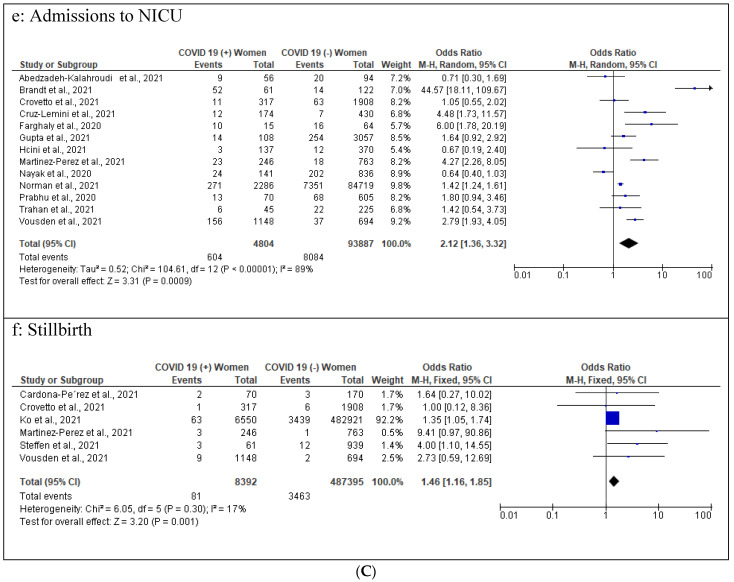
(**A**) Forest plots of adverse maternal outcomes. (**a**) Maternal death. (**b**) Preeclampsia. (**c**) Cesarean delivery. (**d**) Miscarriage/abortion. (**e**) PROM/PPROM. (**f**) Operative vaginal birth. PROM, Pre-labor rupture of membrane. PPROM, Preterm pre-labor rupture of membrane. (**B**) Forest plots of adverse fetal outcomes. (**a**) Fetal distress. (**b**) Intrauterine death. (**C**) Forest plots of adverse neonatal outcomes. (**a**) Preterm birth. (**b**) Low Birth weight. (**c**) Fifth minute Apgar score <7. (**d**) Neonatal death. (**e**) Admissions to NICU. (**f**) Stillbirth. NICU, Neonatal intensive care unit.

The reported maternal outcomes included maternal deaths, miscarriages/abortions, preeclampsia, PROMs/PPROMs, cesarean deliveries, and operative vaginal births. Out of these outcomes, maternal deaths, preeclampsia and cesarean deliveries were found to be statistically significant. In terms of studies on maternal deaths, two studies [24,33] reported an increased risk with COVID-19 during pregnancy. Ten studies [14,17,19,21,22,24,27,30,33,34] reported data on maternal death, and five of them were excluded [14,17,19,22,34] from the meta-analysis because no maternal death was reported in one or both arms. Meta-analysis of the remaining five studies (7953 COVID-19 infected versus 489,454 COVID-19 non-infected) revealed a significant increase in maternal death among COVID-19 infected pregnant women (pooled OR 7.05 [95% CI 2.41−20.65]; *p* < 0.05; I^2^ = 72 %). Based on 16 studies (10,050 COVID-19 infected pregnancies and 497,036 COVID-19 non-infected pregnancies) [14,15,16,17,18,19,21,22,24,26,29,31,32,33,34], a significant increase in preeclampsia during pregnancy was identified among women in the infected pregnant cohort compared to the non-infected comparator (pooled OR 1.39 [95% CI 1.29−1.50]; *p* < 0.05; I^2^ = 25 %). Out of 21 included studies, 20 studies (12,982 COVID-19 infected pregnancies and 583,619 COVID-19 non-infected pregnancies) [14,15,16,17,18,19,20,21,22,23,24,25,26,27,28,29,31,32,33,34] provided data on cesarean section and found a statistically significant increase in cesarean section deliveries among infected women (pooled OR 1.67 [95% CI 1.29−2.15]; *p* < 0.05; I^2^ = 95%). Only two studies provided data on termination of pregnancy [14,22], but no meta-analysis was carried out as there was no termination of pregnancies in the non-infected cohort of one of the studies [14]. Four studies reported miscarriages/abortions [15,18,27,34], and no statistically significant difference of miscarriages/abortions was found between COVID-19 infected and non–infected pregnant women (pooled OR 1.56 [95% CI 0.59−4.12]; *p* = 0.37; I^2^ = 68 %). Pooled odds of 1358 COVID-19 infected pregnancies and 4045 non-infected pregnancies [14,17,19,26,31,32,33] revealed no statistically significant difference of PROM/PPROM between COVID-19 infected and non-infected pregnancies (pooled OR 1.25 [95% CI 0.85−1.84]; *p* = 0.25; I^2^ = 65%) (Figure 4A).

#### 3.7.2. Adverse Fetal Outcomes

The reported fetal outcomes included intrauterine death and fetal distress. Out of these, fetal distress was found to be statistically significant. Based on the data from 1248 newborns born to COVID-19 infected pregnant women and 7422 newborns born to COVID-19 non-infected pregnant women [14,18,21,31,33], a statistically significant increase in fetal distress was observed among the newborns of the COVID-19 infected women compared to the COVID-19 non-infected (pooled OR 1.66 [95% CI 1.35−2.05]; *p* < 0.05; I^2^ = 26%). Four studies [16,17,22,27] reported data on intrauterine death, and of them, one study [16] was excluded from the meta-analysis as no adverse events were reported in infected and non-infected cohorts. The meta-analysis of the remaining three studies (348 COVID-19 infected pregnancies and 1376 COVID-19 non-infected pregnancies) found no statistically meaningful change in intrauterine deaths related to COVID-19 infection during pregnancy (pooled OR 1.79 [95% CI 0.51−6.23]; *p* = 0.36; I^2^ = 68%) (Figure 4B).

#### 3.7.3. Adverse Neonatal Outcomes

The reported neonatal outcomes included preterm birth, low birth weight, stillbirth, fifth minute Apgar score < 7, admissions to NICU, and neonatal death. All of these outcomes were found to be statistically significant, except neonatal death. Pooled preterm birth [14,15,16,17,18,19,20,21,22,23,24,25,26,29,31,32,33,34] of 10,555 births to COVID-19 infected women compared to 498,064 COVID-19 non-infected in 18 studies (pooled OR 1.86 [95% CI 1.34−2.58]; *p* < 0.05; I^2^ = 90%); pooled low birth weight [14,32,33] of 807 births to COVID-19 infected women compared to 1743 to COVID-19 non-infected women in three studies (pooled OR 1.69 [95% CI 1.35−2.11]; *p* < 0.05; I^2^ = 0%); pooled fifth minute APGAR score of less than 7 [21,22,26,28] for 2777 births to COVID-19 infected women compared to 88,909 COVID-19 non-infected in four studies (pooled OR 1.44 [95% CI 1.11−1.86]; *p* < 0.05; I^2^ = 0%) and pooled admissions to NICU [14,16,18,19,20,21,22,26,27,28,29,32,34] of 4804 COVID-19 births to infected women compared to 93,887 COVID-19 non-infected in 13 studies (pooled OR 2.12 [95% CI 1.36−3.32]; *p* < 0.05; I^2^ = 89%) were observed to be significantly higher. Nine studies [15,17,18,19,24,25,26,31,34] reported data on stillbirths, but only six studies (8392 in COVID-19 infected pregnancies compared to 487,395 in COVID-19 non-infected) were included in the meta-analysis due to no stillbirths in COVID-19 infected cohorts in two studies [15,25] and no stillbirths in the COVID-19 non-infected cohort in one study [19]. The pooled odds of six included studies revealed a statistically significant increase in stillbirths among COVID-19 infected women compared to that among the COVID-19 non-infected (pooled OR 1.46 [95% CI 1.16−1.85]; *p* = 0.05; I^2^ = 17%). Of the included studies, neonatal deaths were assessed in seven studies [14,16,21,22,26,31,34], but only three studies were eligible for the meta-analysis [16,21,34]. The pooled odds ratio of three studies (1317 births to COVID-19 infected mothers and 3873 births to COVID-19 non-infected) revealed no significant difference in neonatal deaths between COVID-19 infected and non-infected cohorts (pooled OR 1.73 [95% CI 0.60−5.00]; *p* = 0.31; I^2^ = 0%) (Figure 4C).

### 3.8. Comorbidities among COVID-19 Pregnant Women

Fifteen studies [14,15,16,17,18,23,24,25,26,29,30,33,34] reported on pre-gestational diabetes, but two studies [21,22] were excluded from the meta-analysis due to no events in COVID-19 infected pregnancies and 494,282 COVID-19 non-infected pregnancies. 

**Figure 5:**
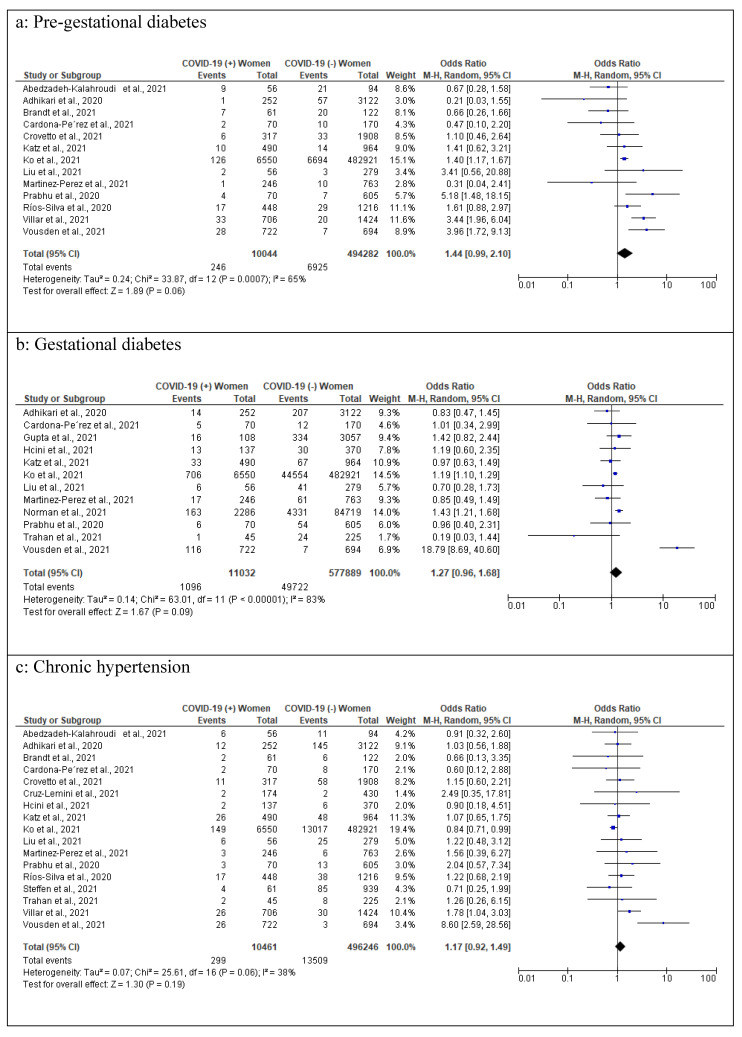

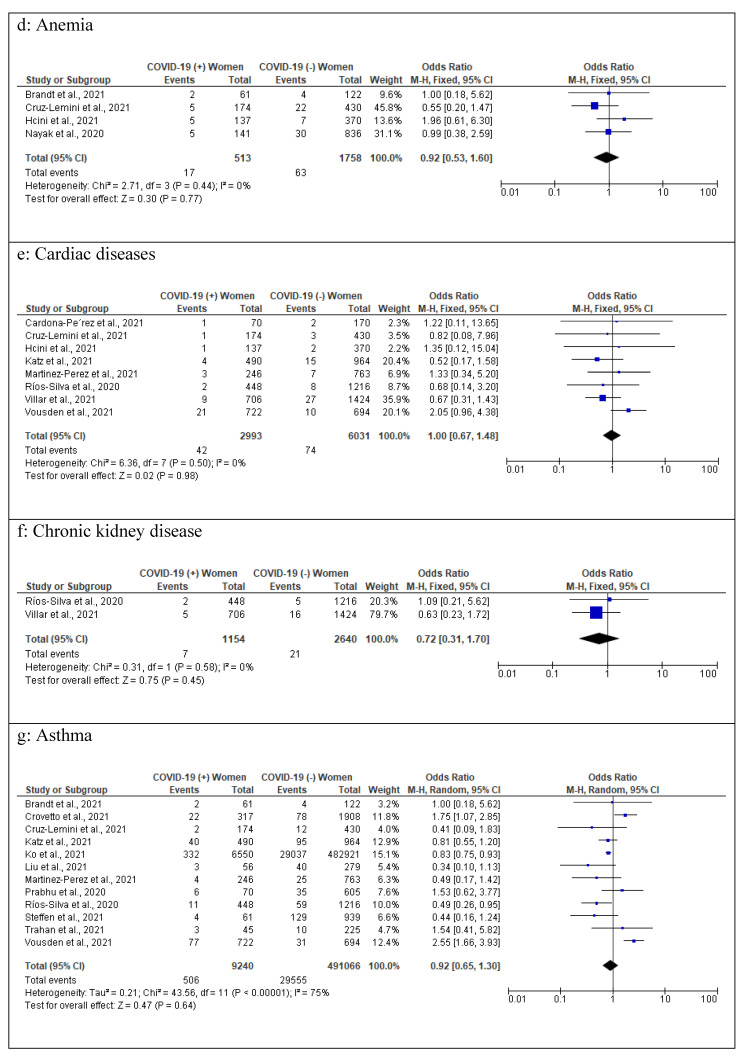

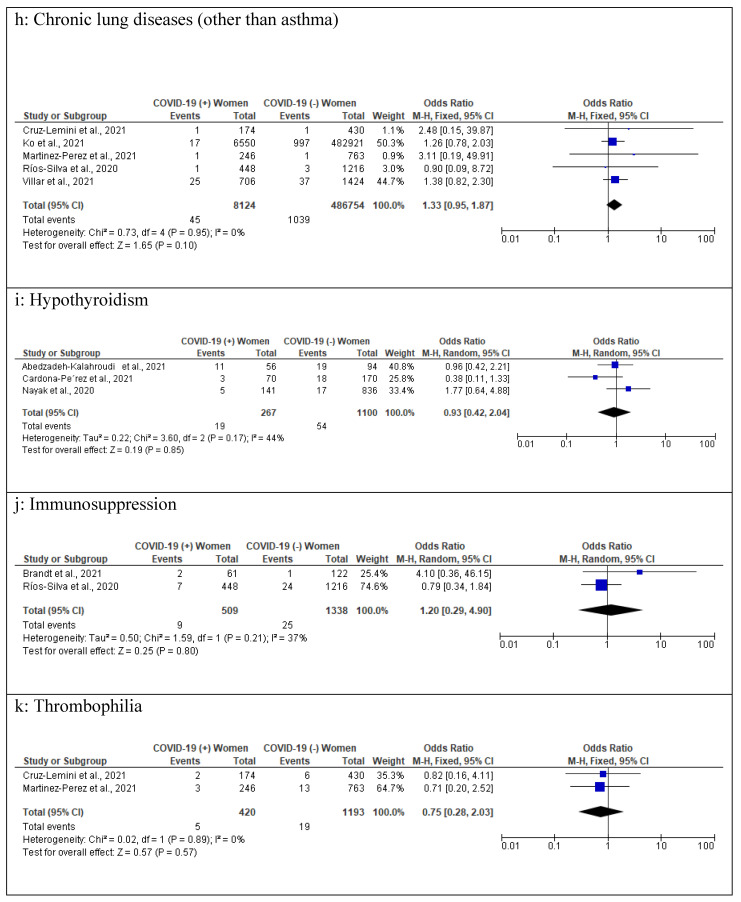
Forest plots of comorbidities among COVID-19 infected women. (**a**) Pre-gestational diabetes. (**b**) Gestational diabetes. (**c**) Chronic hypertension. (**d**) Anemia. (**e**) Cardiac diseases. (**f**) Chronic kidney disease. (**g**) Asthma. (**h**) Chronic lung diseases (other than asthma). (**i**) Hypothyroidism. (**j**) Immunosuppression. (**k**) Thrombophilia.

A higher but non-significant increase in pre-gestational diabetes was observed in infected women compared to non-infected women (pooled OR 1.44 [95% CI 0.99−2.10]; *p* = 0.06; I^2^ = 65%). Gestational diabetes [15,17,21,22,23,24,25,26,28,29,32,34] (11032 COVID-19 infected pregnancies and 577,889 COVID-19 non-infected in 12 studies; pooled OR 1.27 [95% CI 0.96−1.68]; *p* = 0.09; I^2^ = 83%) was also high among COVID-19 infected women compared to COVID-19 non-infected; however, the difference still was not significant. Pooled odds of 10,461 COVID-19 infected pregnancies and 496,246 COVID-19 non-infected in 17 studies revealed no statistically significant difference in chronic hypertension [14,15,16,17,18,19,22,23,24,25,26,29,30,31,32,33,34] (pooled OR 1.17 [95% CI 0.92−1.49]; *p* = 0.19; I^2^ = 38%) between COVID-19 infected and non-infected pregnant women. Data on asthma were reported by 14 studies, but only 12 studies [16,18,19,23,24,25,26,29,30,31,32,34] were included in the meta-analysis (9240 COVID-19 infected pregnancies and 491,066 COVID-19 non-infected pregnancies) as there were no events reported either in infected or non-infected women in two studies [17,22]. The pooled odds ratio of the 12 included studies revealed no risk of being infected with COVID-19 due to asthma during pregnancy (pooled OR 0.92 [95% CI 0.65−1.30]; *p* = 0.64; I^2^ = 75%). Similarly, no statistically significant differences between COVID-19 infected and non-infected pregnancies were observed with relevance to anemia [16,19,22,27] (513 COVID-19 infected pregnancies versus 1758 COVID-19 non-infected pregnancies; four studies; pooled OR 0.92 [95% CI 0.53−1.60]; *p* = 0.77; I^2^ = 0%), cardiac diseases [17,19,22,23,26,30,33,34] (2993 COVID-19 infected pregnancies versus 6031 COVID-19 non-infected; eight studies; pooled OR 1.00 [95% CI 0.67−1.48]; *p* = 0.98; I^2^ = 0%), chronic kidney disease [30,33] (1154 COVID-19 infected pregnancies versus 2640 COVID-19 non-infected; two studies; pooled OR 0.72 [95% CI 0.31−1.70]; *p* = 0.45; I^2^ = 0%), chronic lung diseases other than asthma [19,24,26,30,33] (8124 infected pregnancies versus 486,754 COVID-19 non-infected; five studies; pooled OR 1.33 [95% CI 0.95−1.87]; *p* = 0.10; I2 = 0%), hypothyroidism [14,17,27] (267 COVID-19 infected pregnancies versus 1100 COVID-19 non-infected; three studies; pooled OR 0.93 [95% CI 0.42−2.04]; *p* = 0.85; I2 = 44%), immunosuppression [16,30] (509 COVID-19 infected pregnancies versus 1338 COVID-19 non-infected; two studies; pooled OR 1.20 [95% CI 0.29−4.90]; *p* = 0.80; I2 = 37%) and thrombophilia [19,26] (420 COVID-19 infected pregnancies versus 1193 COVID-19 non-infected; two studies; pooled OR 0.75 [95% CI 0.28−2.03]; *p* = 0.57; I2 = 0%) (Figure 5).

## 4. Discussion

We conducted this systematic review to pool the available evidence of adverse perinatal outcomes caused by COVID-19 infection in pregnancy. We retrieved a total of 21 observational studies that assessed the adverse perinatal outcomes in pregnant women with COVID-19 infection published from December 2019 to June 2021. 

Overall findings of our study were, (1) the reported incidence rates of COVID-19 infection among pregnant women ranged from 1.3% to 27%, disregarding the fact that the results were based on single-center studies to multinational studies; (2) with regards to the adverse maternal outcomes, we found that there was a statistically significant increase in maternal deaths, preeclampsia, and cesarean deliveries, while miscarriages/abortions, PROMs/PPROMs, and operative vaginal births were non-significant in COVID-19 infected pregnant women compared to non-infected; (3) with regards to the adverse fetal outcomes, fetal distress was found to be statistically significant, while intrauterine death was non-significant in COVID-19 infected pregnancies; and (4) with regards to the adverse neonatal outcomes, all reported fetal outcomes except neonatal death, including preterm birth, low birth weight, stillbirth, fifth minute Apgar score < 7, and admissions to NICU showed significant differences in births to COVID-19 infected women compared to non-infected.

The current study findings were consistent with previously published systematic reviews relevant to maternal death [35], preeclampsia [36], preterm birth [35,36], stillbirth [36] and admissions to NICU [35]. In addition to those findings, we found increased cesarean section deliveries among COVID-19 infected women compared to non-infected, 12982, and 583619. However, the data included in the present study did not consider whether those cesarean sections were elective or emergency cases based on COVID-19 status. Pooling of comorbidity data of infected and non-infected pregnant women revealed that comorbidities during pregnancy were not significantly higher in COVID-19 infected pregnancies. This finding was inconsistent with the findings of a previous systematic review, which observed a higher risk of COVID-19 infection in pregnancy when having pre-gestational diabetes mellitus, gestational diabetes mellitus, and chronic hypertension [35]. Out of 21 studies, more than 90% of the studies in this review assessed perinatal outcomes regardless of the disease severity. Consequently, not enough information was available to assess the differences in maternal, fetal, and neonatal outcomes based on disease severity. Therefore, further studies are recommended to assess the perinatal outcomes based on disease severity in order to clear up uncertainties in this area.

### 4.1. Implications for Clinical Practice

Healthcare providers should be aware that women infected with COVID-19 have an elevated risk of disease severity, including maternal mortality. Pregnant women should be advised of the disease’s increased severity and encouraged to take precautions to avoid infection. Primary healthcare providers will need to balance the necessity for routine multidisciplinary prenatal care and the management of women suspected of having COVID-19 infection, preferably via virtual antenatal clinics. Pregnant women who become infected with COVID-19 before reaching term may require management in a tertiary healthcare facility equipped with cesarean section and NICU facilities to manage preterm infants, infants with low Apgar scores, and infants with fetal distress.

### 4.2. Strengths and Limitations

This systematic review has several strengths. First, the study followed a sound methodology and was able to quantify the findings using meta-analyses. Second, a comprehensive search strategy was used to minimize the risk of missing relevant studies. Third, the screening was independently assessed by pairs of reviewers, and discrepancies solved by consensus. Fourth, excluding the publication types such as case studies, case reports, and case series left studies with a quality study design included in the final analysis. Finally, the present systematic review adhered to a rigorous quality appraisal. An important amount of evidence was summarized and critically appraised in addition to the highlighted evidence gaps.

Our systematic review also has limitations. Firstly, the method of diagnosis of COVID-19 in pregnancy was different from study to study. Secondly, without data on disease severity, perinatal outcomes based on disease severity could not be determined. Thirdly, many studies represented developed countries with only meager contributions from low-resource countries. However, the findings of this systematic review have implications for low and middle-income countries with limited resources, where the negative impacts are prominent due to region-specific management strategies and resources. Finally, asymmetry of the funnel plots was observed for the assessed variables, and the presence of publication bias was suggested. This asymmetry may be also due to some other factors such as poor methodological design, reporting bias, chance or study heterogeneity. Despite all limitations, we undertook a comprehensive literature review and meta-analysis with the most updated findings relevant to adverse perinatal outcomes in COVID-19 infected pregnant women.

## 5. Conclusions

Several adverse maternal, fetal, and neonatal effects were significantly higher in COVID-19 infected pregnant women than non-infected. These included maternal death, preeclampsia, cesarean section delivery, fetal distress, preterm birth, low birth weight, stillbirth, low Apgar score at the fifth minute, and admission to NICU. The comorbidity conditions had no added risk of being infected with COVID-19 infection during pregnancy. Therefore, a COVID-19 infected pregnant woman should be treated with special precautions to avoid and minimize the identified adverse events during perinatal care. Further studies are recommended to collect more robust data relevant to the adverse perinatal outcomes that will enable effective clinical decision-making in maternal and child health care.

## Figures and Tables

**Table 1:** Characteristics of included studies.

Study	Country	Study Design	Study Population	Sample Size		Data Collection Period
				COVID-19 (+) Pregnant Women	COVID-19 (−) Pregnant Women	
Abedzadeh-Kalahroudi et al., 2021 [14]	Iran	Prospective cohort study	Single-center	56	94	March to November 2020
Adhikari et al., 2020 [15]	USA	Prospective cohort study	Single-center	252	3122	18 March to 22 August 2020
Brandt et al., 2021 [16]	USA	Case–control study	Single-center	61	122	11 March to 11 June 2020
Cardona-Pe’rez et al., 2021 [17]	Mexico	Case–control study	Single-center	70	170	22 April to 25 May 2020
Crovetto et al., 2021 [18]	Spain	Prospective cohort study	Multicenter	317	1908	15 March to 31 May 2020
Cruz-Lemini et al., 2021 [19]	Spain	Prospective cohort study	Multicenter	174	430	23 March to 31 May 2020
Farghaly et al., 2020 [20]	USA	Retrospective cohort study	Single-center	15	64	March to May 2020
Gupta et al., 2021 [21]	India	Retrospective cohort study	Single-center	108	3057	1 September to 30 November 2020
Hcini et al., 2021 [22]	France	Prospective cohort study	Single-center	137	370	16 June to 16 August 2020
Katz et al., 2021 [23]	USA	Case–control study	Multicenter	490	964	19 March to 31 May 2020
Ko et al., 2021 [24]	USA	Retrospective cohort study	Multicenter	6550	482,921	March to September 2020
Liu et al., 2021 [25]	USA	Retrospective cohort study	Single-center	56	279	10 April to 10 June 2020
Martinez-Perez et al., 2021 [26]	Spain	Prospective cohort study	Multicenter	246	763	23 March to 31 May 2020
Nayak et al., 2020 [27]	India	Retrospective cohort study	Single-center	141	836	1 April to 15 May 2020
Norman et al., 2021 [28]	Sweden	Prospective cohort study	Nationwide	2286	84,719	11 March 2020 to 8 March 2021.
Prabhu et al., 2020 [29]	USA	Prospective cohort study	Multicenter	70	605	22 March to 20 April 2020
Ríos-Silva et al., 2020 [30]	Mexico	Retrospective cohort study	Nationwide	448	1216	28 February to 25 May 2020
Steffen et al., 2021 [31]	USA	Prospective cohort study	Multicenter	61	939	1 May to 22 September 2020
Trahan et al., 2021 [32]	Canada	Cohort study	Multicenter	45	225	22 March to 31 July 2020
Villar et al., 2021 [33]	Argentina, Brazil, Egypt, France, Ghana, India, Indonesia, Italy, Japan, Mexico, Nigeria, North Macedonia, Pakistan, Russia, Spain, Switzerland, UK, US	Prospective cohort study	Multinational	706	1424	2 March to October 2020
Vousden et al., 2021 [34]	United Kingdom	Prospective cohort study	Nationwide	1842	1148	1 March to 31 August 2020

**Table 2:** Characteristics of COVID-19 infected pregnant women.

Study	Age (Years) ^a^	Parity	Gestational Age at Delivery (Weeks) ^a^
Abedzadeh-Kalahroudi et al., 2021 [14]	31.6 (6.1)	Primiparous: 33.9%	37.1 (3.1)
Adhikari et al., 2020 [15]	27.0 (6.6)	Nulliparous: 29%	Range <34 wk to ≥40 wk
Brandt et al., 2021 [16]	30.3 (6.4)	Median (IQR): 2 (1–3)	Mild symptomatic group: 39.0 ± 2.7; Severe symptomatic group: 34.0 ± 5.8
Cardona-Pe’rez et al., 2021 [17]	Median: 26 Range: 13–45	Median: 0; Range 0–3	Median (IQR) 38.1 (36.3–39.3)
Crovetto et al., 2021 [18]	Median (IQR): 33.3 (29–37)	Nulliparous: 53%	39.1 (2.1)
Cruz-Lemini et al., 2021 [19]	32.6	Nulliparous: 38%	39.0
Farghaly et al., 2020 [20]	Mean: 33.4	NR	NR
Gupta et al., 2021 [21]	24.7 (2.4)	Nulliparous: 41.6%	36.6 (3.3)
Hcini et al., 2021 [22]	Median (IQR): 25 (21–31)	Median (IQR): 2 (1–5)	NR
Katz et al., 2021 [23]	30.4 (6.2)	Parity 0: 37.5%; Parity 1: 28.3%; Parity 2+: 34.2%	38.1 (2.6)
Ko et al., 2021 [24]	Median: 28.0 Range: 13–49	NR	NR
Liu et al., 2021 [25]	30.3 (6.4)	Median (IQR): 1 (0–2)	Median (IQR): 39 (38–40)
Martinez-Perez et al., 2021 [26]	32.6	Nulliparous: 38.5%	38.6
Nayak et al., 2020 [27]	Range: <20 to >30	Primiparous: 39%	NR
Norman et al., 2021 [28]	31.4 (5.0)	Nulliparous: 43.1%	39.2 (2.1)
Prabhu et al., 2020 [29]	NR	NR	NR
Ríos-Silva et al., 2020 [30]	Median (IQR): 29 (25–33)	NR	NR
Steffen et al., 2021 [31]	Median (IQR): 28 (24–32)	NR	Median (IQR) 39 (37.1–39.6)
Trahan et al., 2021 [32]	Range: <25 to 35+	Parity 0: 33%; Parity 1: 27%; Parity 2+: 40%	38.9 (2.2)
Villar et al., 2021 [33]	30.0 (6.1)	NR	37.9 (3.3)
Vousden et al., 2021 [34]	Range: <20 to ≥35	Primiparous: 41.2%	Median (IQR) 39 (38–40)

^a^ Mean ± SD if not mentioned otherwise; SD: Standard deviation, NR: Not reported, IQR: Interquartile range.

**Table 3:** Summary findings of individual studies.

Study	The Outcome of the Study (Comparison of COVID 19 Infected and Non-Infected Pregnant Women) ‡			
	Increased Risk/No Difference	Maternal Risk/s	Fetal Risk/s	Neonatal Risk/s
Abedzadeh-Kalahroudi et al., 2021 [14]	Increased risk	Preeclampsia, cesarean section delivery	Fetal distress	Preterm birth, low Apgar score
Adhikari et al., 2020 [15]	No difference			
Cardona-Pe’rez et al., 2021 [17]	Increased risk	Preeclampsia		
Crovetto et al., 2021 [18] †	No difference			
Cruz-Lemini et al., 2021 [19] ††	Increased risk	Pre-labor rupture of membranes		
Farghaly et al., 2020 [20]	Increased risk	Cesarean section delivery		Low mean Apgar score at the fifth minute
Gupta et al., 2021 [21]	Increased risk	Cesarean section delivery	Fetal distress	Preterm birth, low birth weight, low Apgar score
Hcini et al., 2021 [22]	Increased risk		Intra-uterine death	
Katz et al., 2021 [23]	Increased risk			Preterm birth
Ko et al., 2021 [24]	Increased risk	Maternal death		Preterm birth
Liu et al., 2021 [25]	No difference			
Martinez-Perez et al., 2021 [26]	Increased risk	Pre-labor rupture of membranes		Preterm birth, neonatal intensive care unit admission
Nayak et al., 2020 [27]	Increased risk	Cesarean section delivery		
Norman et al., 2021 [28]	Increased risk			Neonatal intensive care unit admission
Prabhu et al., 2020 [29]	Increased risk	Cesarean section delivery		
Ríos-Silva et al., 2020 [30]	No difference			
Steffen et al., 2021 [31]	No difference			
Trahan et al., 2021 [32]	No difference			
Villar et al., 2021 [33]	Increased risk	Maternal death, preeclampsia		Preterm birth
Vousden et al., 2021 [34]	Increased risk	Cesarean section delivery		Neonatal intensive care unit admission

^‡^ Relevant to the studied perinatal outcomes in the current systematic review, ^†^ No difference in the overall rates but the symptomatic status was associated with modest increases in preterm delivery and intrapartum fetal distress, ^††^ Study encompassed only the asymptomatic pregnant women. One study was not included in the table as its outcome was based on disease severity [17].

## Data Availability

The data presented in this study are available on request from the corresponding author.

## Supplementary Materials

The following are available online at https://www.mdpi.com/article/10.3390/healthcare10020203/s1, Table S1: Medline search strategy used in systematic review and meta-analysis of adverse perinatal outcomes in COVID-19 infected pregnant women: a systematic review and meta-analysis; Table S2: Quality assessment of the included studies based on National Institute of Health’s study quality assessment tool.
