# Supplementary material for: Adverse Perinatal Outcomes in COVID-19 Infected Pregnant Women: A Systematic Review and Meta-Analysis
Source: Healthcare (Basel). 2022 Jan 20;10(2):203. doi: 10.3390/healthcare10020203 (PMC8871986; doi:10.3390/healthcare10020203)
Supplement: Supplementary file 1 [file healthcare-10-00203-s001.zip › healthcare-1518024-supplementary.pdf]

Table S1. Medline search strategy used in systematic review and meta-analysis of adverse perinatal outcomes in COVID-19 infected pregnant women: a systematic review and meta-analysis.

- Perinatal outcomes: antenatal care.mp. OR Prenatal Care/ prenatal care.mp. OR Pregnancy Outcome/ or Pregnancy Complications, Infectious/ OR pregnancy outcome*.mp. OR Pregnancy Complications/ OR (Pregnancy Outcome or Pregnancy Complications).mp. OR Obstetric complication*.mp. OR perinatal outcome*.mp. OR Maternal Mortality/ OR maternal death*.mp. OR maternal mortality.mp. OR pregnancy loss.mp. OR fetal death.mp. OR Fetal Death/ OR Foetal death.mp. OR miscarriage*.mp. OR abortion*.mp. OR termination of pregnancy.mp. OR Abortion, Induced/ OR Abortion, Threatened/ OR Abortion, Spontaneous/ OR Abortion, Missed/ OR preterm birth.mp. OR Premature Birth/ OR Obstetric Labor, Premature/ OR preterm labour.mp. OR preterm labor.mp. OR preterm delivery.mp OR premature birth.mp. OR Premature Birth/ OR premature delivery.mp. OR Pre-Eclampsia/ OR premature lab*.mp. OR stillbirth.mp. or Stillbirth/ OR Fetal Death/ OR intrauterine death.mp. OR neonatal death.mp. OR Perinatal Death/ OR Infant Mortality/ OR newborn death.mp. OR perinatal mortality.mp. OR Perinatal Mortality/ OR perinatal death.mp. OR Perinatal Death/ OR cesarean section.mp. OR Cesarean Section/ OR cesarean deliver*.mp. OR instrumental deliver*.mp. OR assisted deliver*.mp. OR assisted labo*.mp. OR operative birth.mp. OR operative deliver*.mp. OR forceps deliver*.mp. OR induction of labo*.mp.
- COVID-19: Coronavirus Infections/ OR Coronavirus.mp. OR Coronavirus/ OR covid-19.mp. OR COVID-19/ OR 2019 nCoV.mp. OR SARS-CoV-2/ OR ("2019" adj3 (corona OR covid OR nCoV)).mp. OR Severe Acute Respiratory Syndrome Coronavirus 2.mp. OR COVID 19 pandemic.mp. OR Novel corona virus.mp.
- Perinatal outcomes AND COVID-19

Table S2. Quality assessment of the included cohort studies based on National Institute of Health's study quality assessment tool.

**Study**	**1. Was the research question or objective in this paper clearly stated?**	**2. Was the study population clearly specified and defined?**	**3. Was the participation rate of eligible persons at least 50%?**	**4. Were all the subjects selected or recruited from the same or similar populations (including the same time period)? Were inclusion and exclusion criteria for being in the study prespecified and applied uniformly to all participants?**	**5. Was a sample size justification, power description, or variance and effect estimates provided?**	**6. For the analyses in this paper, were the exposure(s) of interest measured prior to the outcome(s) being measured?**	**7. Was the timeframe sufficient so that one could reasonably expect to see an association between exposure and outcome if it existed?**	**8. For exposures that can vary in amount or level, did the study examine different levels of the exposure as related to the outcome (e.g., categories of exposure, or exposure measured as continuous variable)?**	**9. Were the exposure measures (independent variables) clearly defined, valid, reliable, and implemented consistently across all study participants?**	**10. Was the exposure(s) assessed more than once over time?**	**11. Were the outcome measures (dependent variables) clearly defined, valid, reliable, and implemented consistently across all study participants?**	**12. Were the outcome assessors blinded to the exposure status of participants?**	**13. Was loss to follow-up after baseline 20% or less?**	**14. Were key potential confounding variables measured and adjusted statistically for their impact on the relationship between exposure(s) and outcome(s)?**
**Abedzadeh-Kalahroudib *et al.*, 2021**	1	1	1	1	0	1	1	2	1	2	1	2	2	0
**Adhikari *et al*, 2020**	1	1	1	1	1	1	1	2	1	2	1	2	2	0
**Liu Andrusier *et al.*, 2021**	1	1	1	1	0	1	2	2	1	2	1	2	2	1
**Vousden Bunch *et al.*, 2021**	1	1	1	0	1	1	1	2	1	2	1	2	2	1
**Crovetto *et al*, 2021**	1	1	1	1	1	1	1	2	1	2	1	0	2	0
**Cruz-Lemini *et al.*, 2021**	1	1	1	1	1	1	1	2	1	2	1	0	2	1
**Ko *et al.*, 2021**	1	1	1	1	1	1	2	2	1	2	1	2	2	1
**Farghaly *et al.*, 2020**	1	1	1	1	0	1	2	2	1	2	1	2	2	0
**Hcini *et al*, 2021**	1	1	1	1	0	1	1	2	1	2	1	2	2	1
**Martinez- *et al., 2021***	1	1	1	1	1	1	1	2	1	2	1	2	0	1
**Norman *et al.*, 2021**	1	1	1	1	1	1	1	2	1	2	1	2	2	1
**Nayak, *et al.*, 2020**	1	1	1	1	0	1	2	2	0	2	1	2	2	0
**Prabhu *et al., 2020***	1	1	1	1	0	1	1	2	1	2	1	2	2	0
**Gupta *et al., 2021***	1	1	1	1	2	1	2	2	1	2	1	2	2	0
**Río-Silva *et al., 2020***	1	1	1	1	0	1	2	2	1	2	1	2	2	0
**Steffen *et al., 2021***	1	1	1	1	0	1	1	2	1	2	1	2	0	0
**Trahan, *et al., 2021***	1	1	1	2	1	1	2	2	0	2	2	2	2	0
**Villar *et al.*, 2021**	1	1	1	1	1	1	1	2	1	2	1	2	0	1

Quality assessment of the included case-control studies based on National Institute of Health's study quality assessment tool.

**Study**	**1. Was the research question or objective in this paper clearly stated?**	**2. Was the study population clearly specified and defined?**	**3. Did the authors include a sample size justification?**	**4. Were controls selected or recruited from the same or similar population that gave rise to the cases (including the same timeframe)?**	**5. Were the definitions, inclusion and exclusion criteria, algorithms or processes used to identify or select cases and controls valid, reliable, and implemented consistently across all study participants?**	**6. Were the cases clearly defined and differentiated from controls?**	**7. If less than 100 percent of eligible cases and/or controls were selected for the study, were the cases and/or controls randomly selected from those eligible?**	**8. Was there use of concurrent controls?**	**9. Were the investigators able to confirm that the exposure/risk occurred prior to the development of the condition or event that defined a participant as a case**	**10. Were the measures of exposure/risk clearly defined, valid, reliable, and implemented consistently (including the same time period) across all study participants?**	**11. Were the assessors of exposure/risk blinded to the case or control status of participants?**	**12. Were key potential confounding variables measured and adjusted statistically in the analyses? If matching was used, did the investigators account for matching during study analysis?**
**Brandt *et al.*, 2021**	1	1	0	1	1	1	2	1	1	1	2	1
**Cardona-Pe´rez *et al.*, 2021**	1	1	0	1	1	1	2	2	1	1	2	1
**Katz, e*t al.*, 2021**	1	1	0	1	1	1	1	1	1	1	2	1
